# Endovascular treatment of genetically linked aortic diseases

**DOI:** 10.1007/s00772-016-0221-z

**Published:** 2017-01-09

**Authors:** D. Böckler, K. Meisenbacher, A. S. Peters, C. Grond-Ginsbach, M. S. Bischoff

**Affiliations:** 1 Klinik für Gefäßchirurgie und Endovaskuläre Chirurgie, Universitätsklinikum Heidelberg, Im Neuenheimer Feld 110, 69120 Heidelberg, Germany; 2 Klinik für Neurologie, Universitätsklinikum Heidelberg, Heidelberg, Germany

**Keywords:** Connective tissue disease, Marfan, Aorta, Endovascular aortic repair, Thoracic endovascular aortic repair, Bindegewebserkrankung, Marfan, Aorta, EVAR, TEVAR

## Abstract

**Background:**

The most important structural proteins of the vascular wall are collagen and elastin. Genetically linked connective tissue diseases lead to degeneration, aneurysm formation and spontaneous dissection or rupture of arteries. The most well-known are Marfan syndrome, vascular Ehlers-Danlos syndrome (type IV), Loeys-Dietz syndrome and familial aortic aneurysms and dissections.

**Objective:**

This review article addresses the current status of endovascular treatment options for important connective tissue diseases.

**Material and methods:**

Evaluation of currently available randomized studies and registry data.

**Results:**

The treatment of choice for patients that are mostly affected at a young age is primarily conservative or open repair. There is only limited evidence for endovascular aortic repair (EVAR) of abdominal aneurysms or thoracic endovascular aortic repair (TEVAR).

**Conclusion:**

The progression of the disease with dilatation leads to secondary endoleaks and high reintervention rates with uncertain long-term results. For this reason, there is currently consensus that EVAR and TEVAR should be limited to justified exceptional cases and emergency situations in patients with genetically linked aortic diseases.

## Zusammenfassung

**Hintergrund:**

Die wichtigsten Strukturproteine der Gefäßwand sind Kollagen und Elastin. Genetisch bedingte Bindegewebserkrankungen führen zu Degeneration und Aneurysmabildung, spontanen Dissektionen oder Rupturen von Arterien. Am bekanntesten sind das Marfan-Syndrom, das Ehlers-Danlos-Syndrom vom vaskulären Typ, das Loeys-Dietz-Syndrom sowie familiäre Aortenaneurysmen und Dissektionen.

**Fragestellung:**

Welchen Stellenwert haben endovaskuläre Behandlungsoptionen in der Therapie aortaler Pathologien auf dem Boden von Bindegewebserkrankungen?

**Material und Methoden:**

Auswertung aktueller randomisierter Untersuchungen und Registerstudien.

**Ergebnis:**

Die Therapie der Wahl der meist bereits in jungem Alter betroffenen Patienten ist primär konservativ bzw. offen chirurgisch. Zur endovaskulären Therapie abdomineller Aneurysmen (EVAR) oder thorakalen Aortenpathologien (TEVAR) gibt es nur wenig Evidenz.

**Schlussfolgerungen:**

Die Progression der genetisch bedingten Grunderkrankung mit konsekutiver Aortendilatation führt zu sekundären Endoleckagen und vermehrten Reinterventionen mit unsicherem Langzeitergebnis. Deshalb besteht derzeit Konsensus, dass EVAR und TEVAR bei genetisch bedingten Aortenerkrankungen auf begründete Ausnahmefälle und Notfallsituationen begrenzt sein sollten.

## Introduction

Over the past 10 years endovascular treatment has become the method of first choice for both infrarenal aortic aneurysms (AAA) and thoracic aortic disease, e.g. aneurysms, Stanford B dissection, intramural hematoma (IMH), penetrating aortic ulcer (PAU) and traumatic rupture. This is supported by numerous randomized studies (e.g. EVAR1, DREAM OVER, INSTEAD and ADSORB) [1–7], as well as large international registry studies (ENGAGE and GREAT) [8, 9]. Endovascular repair has also become established as the first-line approach in the emergency setting of ruptured AAA in many centers with the relevant practical experience [10]. Here again, results from randomized studies (IMPROVE, AJAX, ECAR) are now available and provide valuable data on establishing the indications and choosing the method [11–14]. Establishing the indications for endovascular aortic repair (EVAR) in AAA is guided by conventional aneurysm surgery and allows treatment from an aneurysm diameter of 5 cm. As is well known, aneurysm morphology, comorbidities, patient treatment wishes and operator or center-specific perioperative mortality and morbidity rates influence the choice of treatment. In contrast, establishing the indications for thoracic endovascular aortic repair (TEVAR) in the heterogeneous group of thoracic aortic diseases depends on the diameter in the case of asymptomatic thoracic aortic aneurysm (TAA) and PAU and, in the case of complex type B dissections or IMH, on symptoms, the presence of organ complications or imaging predictors of rapid progression [15].

Technical and clinical success is based in the long and the short term on adequate and “healthy” landing zones, both proximal and distal to the aortic pathology, for the planned endograft. Higher reintervention rates and increased mortality are known and have been described following EVAR in which the instructions for use (IFU) were not fully observed [16].

Technical and clinical success depends on adequate landing zones

Age and gender also impact treatment outcomes. For example, females with aortic dissection exhibit a higher complication rate in the spontaneous course as well as postoperatively. The Cleveland working group led by Ourielund and Greenberg was able to show the effect of gender on the outcome of EVAR in their own patient population (*n* = 704, 606 males, 86.1%) [17]. Although females had somewhat smaller AAA (5.2 vs. 5.4 cm), both groups were comparable in terms of age and comorbidities. No gender-specific differences were observed in terms of 30-day mortality or in the mean follow-up in terms of migration, reintervention or conversion rate. Other overview articles, however, reported higher long-term mortality rates among females with AAA 5 years following EVAR [18]. Against this backdrop, genetic aortic diseases, such as Marfan or Ehlers-Danlos syndromes, play a particularly prominent role. These patient groups are young, experience syndrome-specific multimorbidity and the aortic disease shows greater progressive dilatation. The aim of this overview article is to briefly discuss the most important aortic diseases through the prism of endovascular treatment options and the importance.

## Genetic aortic diseases

The following provides a short overview of the four best-known diseases or disease complexes involving impaired vascular or aortic wall integrity due to genetic connective tissue defects. As such, they are referred to as connective tissue diseases (CTD). The description of diseases in this article makes no claim to be exhaustive and the reader is referred to further literature [19–21].

### Marfan’s syndrome (MS)

With a prevalence of 1 in 10,000 individuals Marfan’s syndrome, an autosomal dominant disorder first clinically described in 1896 by Antonin-Bernard Marfan and genetically confirmed in the early 1990s, is caused by a mutation of the fibrillin-1 gene (*FBN1*), which maps to chromosome 5q21.1. The Ghent criteria, as well as complementary genetic tests that have become limited in their practicability due to the now >600 different mutations, are used for the typing and confirmation of Marfan’s syndrome [21]. In addition to the eyes and musculoskeletal system, large lumen arteries are particularly affected. Involvement of the cardiac valve and aortic root as well as the risk of aortic dissection and rupture associated with dilatation, crucially affect the prognosis. Dilatation of the sinus of Valsalva already begins in intrauterine life during the embryonic period. There are no absolute diameters on which to base the indications for surgery in children and adolescents. Growth of >1 cm/year or severe valve insufficiency and a Z score (statistical value with respect to mean value and standard deviation in aortic root diameter) >2–3 provide orientation. Surgery is recommended in adults with an aortic root diameter of 50 mm and above [21]. Isolated infrarenal aortic involvement is not known. Thanks to early identification and treatment, the mean life expectancy of patients with Marfan’s syndrome can now be increased to as much as 60 years [22].

### Vascular type (type IV) Ehlers-Danlos syndrome (EDS)

Ehlers-Danlos syndrome (EDS) is a heterogeneous, genetically linked and generally autosomal dominant disorder of collagen synthesis for which 10 subtypes have been identified (Table 1). The most common subtypes are classical types I and II, which are rarely associated with vascular complications. Vascular type IV, caused by defective type III procollagen, is relevant in vascular surgery (prevalence 1:100,000–1:250,000). The defect, encoded by the *COL3A1* gene, results in extreme vascular fragility. Detailed genetic and diagnostic information can be found in works by Superti-Furga et al. [23] and Beighton et al. [24]. Due to vascular rupture, the most common cause of death, the life expectancy of affected individuals is markedly curtailed at 48 years on average. Vascular rupture, which more commonly affects medium-sized arteries, in contrast to Marfan’s syndrome, can occur irrespective of diameter. Pepin et al. published a series of 220 type IV EDS patients, 89% of which experienced complications at the age of 40 years. Mortality was 60% (60% aortic rupture; 15% organ rupture, in particular heart, uterus, spleen, and liver; 7% intracranial hemorrhage; 12% hemorrhage of unknown etiology). True aneurysms are rare in EDS IV [25, 26].

**Table 1:** Subtypes of Ehlers-Danlos syndrome

Nomenclature	Type	Clinical symptoms	Mode of inheritance
Classical	I, II	Rarely vascular complications	AD
Hypermobility	III	Arthritis	AR
Vascular	IV	Rupture of arteries, uterus, intestines, skin	AD
Kyphoscoliosis	VI A, VI B	Hypotonia, osteoporosis, kyphoscoliosis, rupture of arteries or ocular globe	AR
Arthrochalasia	VII A, VII B	Hip subluxation, osteoporosis	AD
Dermatosparaxis	VII C	Doughy, loose skin	AR
Other	V	Loose, hyperelastic skin	X-chromosomal
	VIII	Periodontitis	AD
	IX	Loose skin, osteoporosis	X-chromosomal
	X	Petechiae	?

*AD* autosomal dominant, *AR* autosomal recessive

### Loeys-Dietz syndrome (LDS)

Loeys-Dietz syndrome (LDS) is an aortic syndrome characterized by aortic aneurysm formation associated with marked vascular tortuosity, craniofacial abnormalities and bifid uvula. It is caused by heterozygous mutations in the genes encoding transforming growth factor beta (TGFbeta) receptors I and II [27]. The LDS is clinically distinct from Marfan’s syndrome in that the aortic root may rupture and dissect in early childhood and at a small diameter. Establishing a family history and screening are crucial. As with EDS, mean life expectancy is approximately 22–37 years. Histologically, collagen and elastin abnormalities, triggered by the TGFbeta signaling pathway, are present. For more details, readers are referred to the original works by the author that first described the disorder, Prof. Bart Loeys [28]. Aneurysmal widening of the aorta affects only approximately 9% of LDS patients. A rapid growth rate of 1.8 mm/year in the case of a thoracic localization is worthy of note. Although the infrarenal aorta is less susceptible to aneurysmal lesions, it is generally elongated by a factor of two [29].

In addition to the administration of beta-blockers and possibly also losartan, current recommendations include conventional surgical treatment. The indications for prophylactic surgery need to be made on an individual basis, taking risks and benefits into consideration. On average, vascular repair is necessary at the average age of 16.9 years [22]. In adults, the indication to treat the thoracoabdominal and infrarenal aorta is established at 4.0 cm [21]. Multiple interventions and reinterventions are common in EDS.

### Familial thoracic aortic aneurysms and dissections (FTAAD)

A familial predisposition to thoracic aortic aneurysms and dissections (FTAAD) is seen in 11–19% of cases. So far five gene loci on three different genes have been mapped (*TAAD1*: 5q13–14, *FAA1*: 11q23–24, *TAAD2*: 3p24–25 and *MYH11*: 16p12.2–13.13). Furthermore, alpha-actin mutations (*ACTA2*) are held responsible for 14% of all FTAAD. Media degeneration represents the common pathomechanism. As FTAAD occurs at a young age, it is considered a more aggressive clinical entity [29]. Aneurysms are most frequently localized in the thoracic aorta (66%), followed by AAA (25%) and cerebral aneurysms (8%). Aneurysms and dissections are equally distributed at 50%. The indications to treat aneurysms are based on thresholds in sporadic or non-syndromic aneurysms: thoracic 6.0 cm and infrarenal 5 cm. As with LDS, a rapid growth rate is seen in the familial form (0.21 cm/year) compared with 0.16 cm/year in sporadic aneurysms. Further information on familial abdominal aortic aneurysms (fAAA) can be found in the recommended overview articles by van de Luijtgaarden et al. [30, 31].

## EVAR and TEVAR in genetic aortic disease (GAD)

**Fig. 1:**
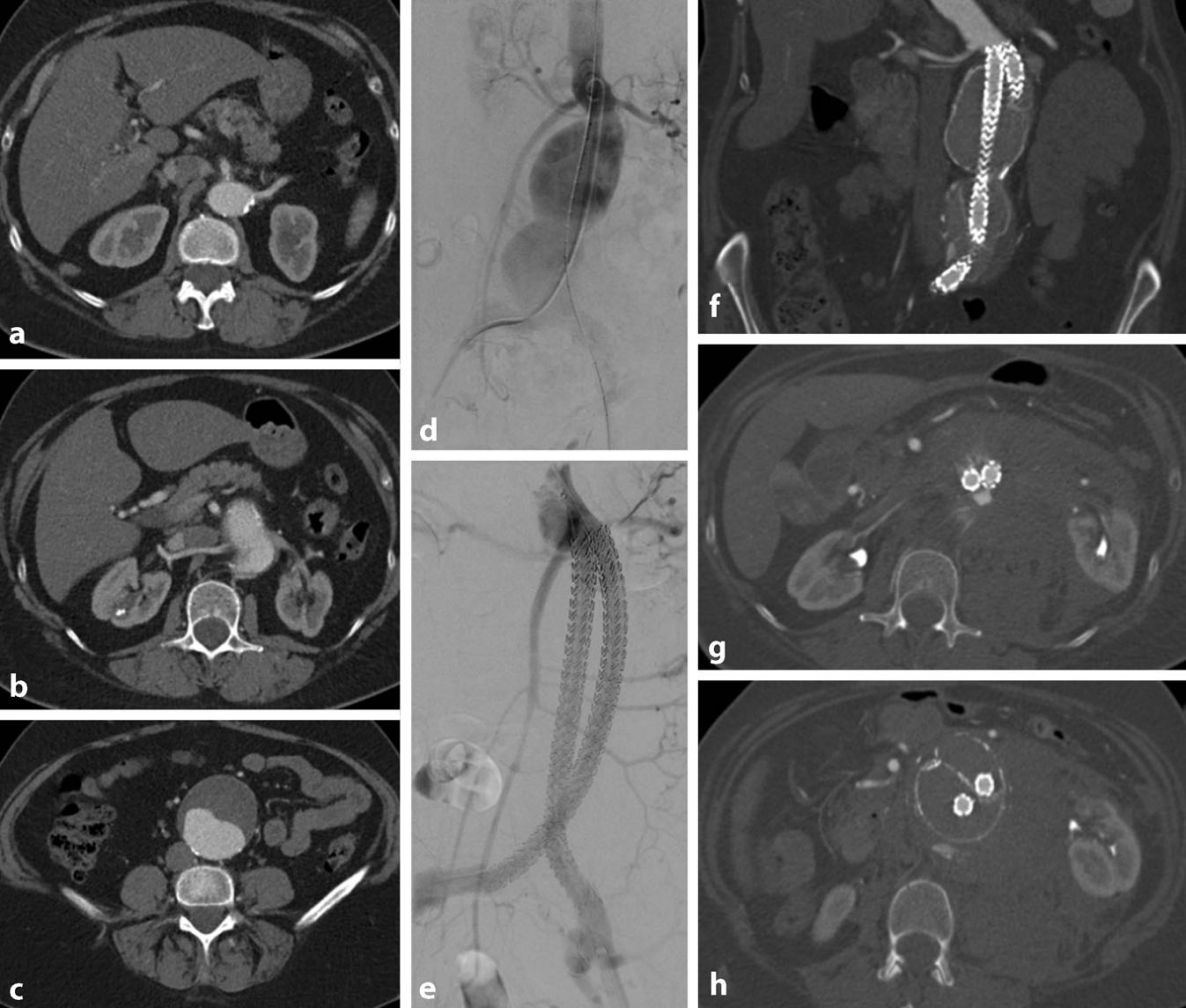
Highly comorbid female patient with suspected type IV Ehlers-Danlos syndrome and 60-mm asymptomatic juxtarenal abdominal aortic aneurysm that was declined for a fenestrated endograft and, due to morphology, untreatable using a chimney technique. Preoperative computed tomography angiography (CTA) shows the origin of the more proximal left renal artery at the level of the superior mesenteric artery (**a**), the juxtarenal start of the aneurysm (**b**) and the maximum transverse diameter (**c**). Intraoperative digital subtraction angiography (DSA) before and after implantation of a Nellix graft showing aneurysm occlusion and no indication of an endoleak (**d, e**). Postoperative CTA at 6 weeks shows a regularly perfused endograft and no indication of migration or type Ia endoleak; however, there is evidence of a type II endoleak in the region of the right distal Nellix limb. Emergency CTA approximately 6 months following implantation shows a secondary-type Ia endoleak (**g**) as well as the dorsally contained rupture with contrast medium extravasation and retroperitoneal hematoma (**h**). Images kindly provided by the Department of Diagnostic and Interventional Radiology, Heidelberg University Hospital

**Fig. 2:**
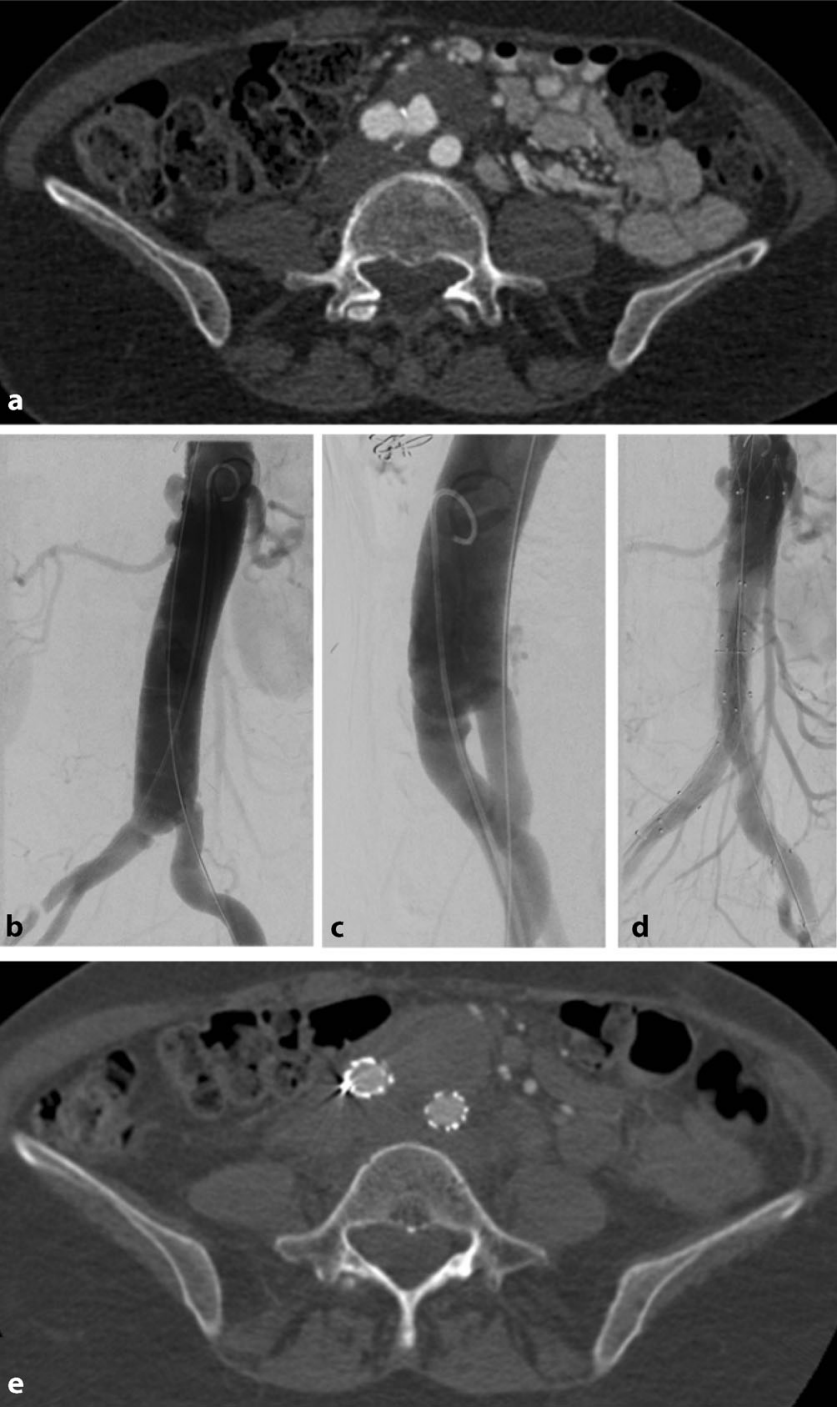
A 48-year-old female patient with Marfan’s syndrome with a contained rupture of a distal anastomotic aneurysm following open thoracoabdominal graft repair. **a** Preoperative CTA shows a distal connecting aneurysm in the region of the aortic bifurcation, status post-aorto-aortic tube interposition using a Coselli graft. **b**–**c** Intraoperative imaging prior to EVAR, anteroposterior and left anterior oblique projections. **d** Control angiography following successful EVAR showing no indication of an endoleak. **e** Postoperative CTA showing complete occlusion of the connecting aneurysm. Images kindly provided by the Department of Diagnostic and Interventional Radiology, Heidelberg University Hospital

In principle, endovascular techniques are not intended for the treatment of the thoracic and abdominal aorta in patients with genetic connective tissue disease. In relevant approval trials, the commercially available endografts were either not investigated in the fragile milieu of the marfanoid aorta or indeed excluded for this indication. The long-term radial force of the endograft, the prerequisite of an anchor zone with sealing properties, cannot be predicted in this patient group. Thus, the application of endovascular techniques in genetic aortic disease (GAD) patients falls a priori outside the IFU (Fig. 1). Milewicz et al. [19] recommend EVAR/TEVAR only for late, chronic pseudoaneurysms of residual native aortic segments in a graft-to-graft approach. Fig. 2 presents a case study with this indication from our own patient population. A 2008 consensus publication, as well as the European Society of Vascular Surgery (ESVS) guidelines due to be published shortly, clearly oppose primary endovascular treatment [20, 32]. In individual cases, patients with a significantly increased risk for an open surgical procedure can be considered for an endovascular approach at a recognized center for the treatment of complex aortic diseases [20, 32]. It is generally accepted in such cases that the indication is given for patients in an emergency setting in whom endovascular treatment represents a life-saving bridging procedure until definitive open repair can be performed. This means that patients can be taken out of the life-threatening situation posed by rupture or the threat of rupture in the case of aortic pain or organ malperfusion while an elective conversion is planned in the interim.

Our own working group reported in 2008 on early clinical experiences in 167 GAD patients, 8 of which underwent TEVAR. Striking features at a mean follow-up of 34 months included a high primary endoleak rate of 38%, a reintervention rate of 25% and, in particular, a rate of disease progression with de novo aneurysms of again 38%. There were no aorta-related deaths [33]. In conclusion, TEVAR was deemed viable as a bridging method due to the low periprocedural mortality rate. A 2015 review conducted by Parisi et al. [34] summarized the largest published series in table form (Table 2 and 3).

**Table 2:** Perioperative results after TEVAR in patients with Marfan’s syndrome

Author	Number of patients	Emergencies *n* (%)	Prior aortic surgery *n *(%)	In-hospital mortality *n* (%)	Paraplegia/ paraparesis *n* (%)	Stroke/TIA *n* (%)	Primary endoleak *n* (%)
Ince et al. [16]	6	ns	5 (83)	0	0	0	0
Nordon et al. [17]	7	3 (42.8)	7 (100)	1 (14)	0	0	ns
Geisbüsch et al. [18]	6	ns	3 (50)	0	0	0	Type I: 1 (16.6)
Botta et al. [19]	12	5 (41.7)	12 (100)	0	0	0	Type I: 1 (8.3)
							Type II: 2 (16.7)
Marcheix et al. [20]	15	2 (13.3)	11 (73)	0	0	1 (6.7)	Type I: 4 (26.7)
							Type II: 1 (6.7)
Waterman et al. [21]	16	3 (18.7)	15 (94)	1 (6.2)	ns	ns	Type I: 3 (18.7)
							Type II: 1 (6.2)
Eid-Lidt et al. [22]	10	10 (100)	5 (50)	1 (10)	0	1 (10)	ns

*ns* not specified, *TIA* transient ischemic attack

**Table 3:** Follow-up results after TEVAR in patients with Marfan’s syndrome

Author	Number of patients	Mean follow-up (months)	Secondary endoleak *n *(%)	New endovascular procedure (*n*)	Open conversion (*n*)	Death (*n*)
Ince et al. [16]	6	51 (12–74)	ns	0	2	1
Nordon et al. [17]	6	16 (3–54)	Type I: 1 (16.7)	2	0	1
			Type III: 1 (16.7)			
Geisbüsch et al. [18]	6	32.8 (3–79)	Type I: 1 (12.5)	1	0	1
			Type III 1 (12.5)			
Botta et al. [19]	12	31 (3–57)	Type I: 1 (8.3)	1	1	0
			Type III: 1 (12.5)			
Marcheix et al. [20]	15	25 (10–59)	Type I: 4 (26.7)	3	5	3
			Type III: 1 (6.7)			
Waterman et al. [21]	15	9 (0–46)	ns	4	7	3
Eid-Lidt et al. [22]	9	59.6 (9–102)	Type I: 1 (22.2)	3	0	1
			Type II: 1 (22.2)			
Total	69	32	13	14	15	10

*ns* not specified

Endovascular treatment in patients with EDS (type IV) primarily comprises coil embolization of supra-aortic branches of the aortic arch or other medium-sized arteries in the context of bleeding [25]. These endovascular occlusion techniques are particularly suited to cases of spontaneous tearing in the visceral artery walls, particularly the hepatic and splenic arteries. A frequent and classical complication of type IV EDS is a carotid cavernous fistula, which presents as insidious, progressive proptosis (exophthalmos) or in association with headaches. Coil embolization can be performed in selected patients if the risk of sacrificing the intracranial internal carotid artery and subsequent stroke can be ruled out at the planning stage using high-resolution imaging techniques.

If there is a relevant predisposition to false aneurysm formation and access vessel rupture (generally the common femoral artery), percutaneous access needs to be considered as opposed to open surgical cut-down, particularly in the case of relevant sheath diameters (>8 French) [35]. Open repair is recommended in the case of access complications of this kind [21]. As there are no large series on EVAR and TEVAR in type IV EDS, it is not possible to make study-based recommendations at this point; however, similar considerations to those in Marfan’s syndrome apply. Due to the fragility of the aortic wall, the questionable durability of endovascular treatment and the resulting higher reintervention rate, extreme caution is advised. Although, in the authors’ opinion, there is a justification for exceptions, such as emergency bridging, these need to be justified on an individual basis, require that patients be provided with detailed information on alternatives if time permits and require careful documentation in the surgical report. It is generally accepted that EVAR and TEVAR should be avoided for type IV EDS [20]. Due to the extremely high risk of injury and rupture, even supposedly simple angiography should be avoided. A study by Cikrit et al. reported a 67% complication rate and a 12% mortality rate for digital subtraction angiography (DSA) [35]. It is possible that future low-profile catheters and endografts may be able to reduce these relevant rates. The use of non-invasive cross-sectional imaging with the possibility of three-dimensional reconstruction is increasingly reducing these risks. Should endovascular treatment nevertheless be necessary, direct suture of the access vessel is recommended.

Endovascular treatment is not recommended in LDS and, apart from individual exceptions is not performed due to the young age of these patients, the rarity of the syndrome and the associated aortic disease. A recent bibliography search (as of 25 July 2016 in MEDLINE/PubMed, https://www.ncbi.nlm.nih.gov/pubmed. nih.gov/pubmed) with “Loeys-Dietz syndrome” and “endovascular therapy” produced only two hits. In 2015, Kalra et al. [36] reported on two patients with LDS and contained ruptures of the descending aorta that were successfully managed by endovascular repair. No follow-up is available. Colby [37] reported on the technically successful treatment of a 23-year-old female patient with a large, dysplastic cavernous intracerebral arterial aneurysm using the Pipeline^TM^ embolization device (Covidien, Medtronic, Santa Rosa, CA). Complete aneurysm occlusion was seen at 10 months following endovascular treatment.

As in Marfan’s syndrome, conservative drug therapy with beta-blockers is initially recommended. Due to the pathophysiological correlations with increased TGFbeta activity in the vessel wall, there appears to be a clear rationale for treatment with losartan. Randomized studies on losartan treatment yielded conflicting results. A recently published meta-analysis by Gao et al. [38] summarized the results from 6 randomized studies of 1398 patients and demonstrated that although losartan treatment significantly reduced aortic root dilatation, no survival benefit was seen compared with the control group. Initial results from small case series on open surgery, particularly on the aortic root, revealed low perioperative mortality rates; however, secondary rupture at other sites was seen during follow-up. Numerous specialist medical societies also recommend specialized interdisciplinary treatment in LDS due the complexity of the disease [39].

There are no large series on EVAR and TEVAR in genetically linked aortic disease

The treatment strategy in FTAAD is complex and non-uniform due to the variable penetrance and expression of the disease and the lack of genotype and phenotype correlation. No endovascular approach has been propagated or published as yet. To date, the available consensus documents have advised against such an approach. This may be attributable to publications from single center studies, among others, showing that patients with fAAA exhibit a high aneurysm-related complication rate. Despite similar aneurysm morphology in 51 out of 255 fAAA patients, van de Luijtgaarden et al. reported a two-fold higher complication rate of 35.3% vs. 19.1% (hazard ratio 2.1, 95% confidence interval 1.2–3.7), a significantly increased reintervention rate (39.2% vs. 20.1%, *p* = 0.004) and greater AAA growth following EVAR (20.8 vs.9.5%, *p* = 0.03). The fact that too little attention is paid in the diagnostic work-up to the possible presence of FTAAD undoubtedly represents a problem in clinical routine.

## Summary

The level of evidence or grade of recommendation on establishing the indications for endovascular treatment of GAD is limited (evidence grade II, level C). The data on technical and clinical treatment outcomes following EVAR and TEVAR in genetically linked aortic disease are also scant. Knowledge and implementation of the recommendations made by specialist societies should form strict requirements. The diagnosis, treatment and follow-up of patients with GAD (e.g. MS, EDS, LDS and TAAD) are complex, challenging and need to be performed in an interdisciplinary approach [26, 40–42]. Thus, the management of GAD should be reserved for specialized centers with appropriate clinical and surgical experience.

## Conclusion

- Vascular surgeons should be aware of the four most important genetically linked vascular and aortic diseases: MS, EDS, LDS and FTAAD.
- Thanks to better and earlier diagnosis, it has been possible to improve life expectancy, at least in Marfan’s syndrome, in recent years.
- Conservative therapy, monitoring and possibly also conventional surgical treatment should be considered standard in the management of GAD.
- Although endovascular repair is primarily not indicated in the sense of a consensus recommendation for elective interventions, it is accepted as a life-saving bridging procedure and justified in individual cases.
- Dilatation behavior and the frequently related tortuosity are causative in the high endoleak and reintervention rates and hence in the less favorable treatment outcomes with EVAR and TEVAR compared with non-syndromic aortic disease.
- Commercially available stent grafts have not been investigated for this indication and, as such, are not approved for the treatment of GAD (use out of IFU).
- As technological advances in stent grafts may have a favorable impact on therapy concepts and treatment outcomes in the future, the significance of endovascular methods for the treatment of GAD requires continuous re-evaluation.
